# The “Island Rule” and Deep-Sea Gastropods: Re-Examining the Evidence

**DOI:** 10.1371/journal.pone.0008776

**Published:** 2010-01-19

**Authors:** John J. Welch

**Affiliations:** Institute of Evolutionary Biology, School of Biological Sciences, University of Edinburgh, Edinburgh, United Kingdom; McGill University, Canada

## Abstract

**Background:**

One of the most intriguing patterns in mammalian biogeography is the “island rule”, which states that colonising species have a tendency to converge in body size, with larger species evolving decreased sizes and smaller species increased sizes. It has recently been suggested that an analogous pattern holds for the colonisation of the deep-sea benthos by marine Gastropoda. In particular, a pioneering study showed that gastropods from the Western Atlantic showed the same graded trend from dwarfism to gigantism that is evident in island endemic mammals. However, subsequent to the publication of the gastropod study, the standard tests of the island rule have been shown to yield false positives at a very high rate, leaving the result open to doubt.

**Methodology/Principal Findings:**

The evolution of gastropod body size in the deep sea is reexamined. Using an extended and updated data set, and improved statistical methods, it is shown that some results of the previous study may have been artifactual, but that its central conclusion is robust. It is further shown that the effect is not restricted to a single gastropod clade, that its strength increases markedly with depth, but that it applies even in the mesopelagic zone.

**Conclusions/Significance:**

The replication of the island rule in a distant taxonomic group and a partially analogous ecological situation could help to uncover the causes of the patterns observed—which are currently much disputed. The gastropod pattern is evident at intermediate depths, and so cannot be attributed to the unique features of abyssal ecology.

## Introduction

The island rule states that after island colonisation, animals undergo predictable patterns of body size evolution, with larger colonising species becoming smaller, and smaller species becoming larger. The result is a graded trend from dwarfism in the largest colonists to gigantism in the smallest [1]–[4]. Because insular habitats are distinctive in a number of ways, this pattern might be variously explained, and a variety of hypotheses have indeed been proposed in the literature [1]–[11]. Recently, McClain et al. [12] made an important advance by testing for analogous patterns of body size evolution in a non-insular system. Specifically, they compared body sizes of animals living in deep-sea benthic habitats with their shallow-water living congeners. Using the Malacolog database version 3.3.3 of Rosenberg [13], McClain et al. [12] took the gastropods of the western Atlantic as a case study, and reported that a highly significant trend from dwarfism to gigantism was evident in the deep-sea species.

From the perspective of understanding the island rule, this result is exciting, because the deep-sea benthos shares some but not all of the ecological characteristics of insular habitats [3]–[5], [12], [14]–[16]; see Discussion. But unfortunately, the debate about the ecological and evolutionary processes is hindered by uncertainty about where, if anywhere, the island rule applies [4], [17]–[20]. In particular, since the appearance of McClain et al.'s study [12], it has been shown that many common tests of the rule are subject to high levels of false positive error [19]–[21]. In particular, if the mainland populations have undergone any body size evolution since the last common ancestor of the genus, then data can appear to manifest the island rule, even if, in reality, there were no differences between patterns of island and mainland evolution. Intuitively, the reason is that when mainland populations have increased in size, they are both more likely to be classified as ‘large’, and more likely to be larger than their insular relatives, regardless of how the latter have evolved. The reverse applied if mainland populations have decreased in size, and together, this can create the misleading impression of a graded trend from dwarfism to gigantism. Price and Phillimore [21] suggested using standardized-major-axis regression to avoid this problem, and Welch [20] suggested a Monte Carlo permutation approach to assess its significance. This test is parametric (*contra* incorrect statements in [20]) but it is distribution-free, and so avoids the assumptions of normality and homogeneity of variance that are unlikely to hold for comparative data [22], [23], and which can also mislead the standard tests.

Here, I re-examine whether deep-sea gastropods manifest the island rule, making use of the improved statistical methods, and data collated from the recently updated Malacolog database [24], which has been both expanded, and revised to reflect advances in gastropod systematics [25]. It is found that the central conclusion of McClain et al. [12] is robust, and that gastropod colonists of the deep-sea benthos do indeed exhibit island-rule-like evolution.

## Methods

All data analysed here were taken from Malacolog version 4.1.1 [24]. This version of the database contains data for non-gastropod Mollusca, but not in sufficient numbers for a statistically robust test, so the present analysis is restricted to gastropods. Details of all species (or in some cases subspecies) with the appropriate measurements were downloaded, yielding 4256 taxa in total. All data are available as Table S1. The measure of body size was the maximum recorded shell length (mm), which was logarithmically transformed. This measure can create artefacts, because its value can depend on sample size - larger samples tending to have larger maximum values [17] - and the raw data necessary to calculate sample means were not available for most of the species represented here. Nevertheless, in the present analysis the use of maxima would cause a bias only if the reported shell lengths do depend strongly on number of measured individuals, and if this number varied with body size in a different way at different depths, i.e., if bigger, but not smaller taxa were under-sampled in deep waters. To classify each species as deep-water, shallow-water or neither, the shallowest and deepest reported depth (m) were used. Following [12], the boundary between the two regions was set at 200m - the average limit of the continental shelf in the Atlantic ocean, and the limit of the oceanic photic zone, capable of sustaining photosynthesis [12], [15], [16], [26]. As such, species were classified as “shallow” if the chosen measure of their depth range was ≤200m (various measures were used; see Results). Only genera that contained both deep and shallow members were retained for further analysis, and each data point consisted of a shallow and deep size estimate for a different genus - in most cases, the mean of the log body sizes of all deep or shallow congeners (taking means after log transformation to avoid bias). Analyses were also repeated after equalizing the number of deep and shallow species in each genus. This was done because most genera contained fewer deep than shallow species, and a smaller number of species will be associated with a smaller sample variance, which could be falsely taken to imply a genuine narrowing in the distribution of body sizes in deep seas, as predicted by the island rule. To equalize the numbers of deep and shallow species, I excluded species so as to maximize the difference in the depth range midpoints (i.e., the mean of the maximum and minimum recorded depths) between the deep and shallow species; for example, in a genus with two deep and five shallow species, I removed the three shallow species with the deepest range midpoints. This is equivalent to the common practice in the island rule literature of retaining the species from the smallest island when multiple equally close insular relatives of a mainland taxon are available (see e.g., [27]). The procedure should maximise the chances of observing an effect, without introducing a bias under the null. The alternative strategy of excluding species at random introduces the problem of combining *p*-values from multiple non-independent tests.

To analyse the data, the deep-sea body sizes for each genus were regressed onto the shallow-water body sizes. Under the null hypothesis of no effect of deep-sea colonization, these two values should be equal on average, and so the best-fit line should have a slope of one. A shallower slope is consistent with the island rule, and represents a narrowing of the distribution of body sizes in the deep-sea species [3]. To obtain the best-fit slope, standardized-major-axis regression was used [21], [28]. The test statistic for this type of regression is the correlation coefficient between *x*+*y* and *x*−*y* (where *x* is the shallow-water body size and *y* is the deep-sea body size). Significance was assessed by randomly permuting the labels for the deep and shallow species within each genus 100,000 times, to generate a null distribution of the test statistic. The *p*-value was calculated as the proportion of these randomized coefficients that were equal to or more extreme in value than the true test statistic, doubled for a two-tailed test [20]. To replicate the method of [12], an ordinary-least-squares regression was also carried out, calculating significance with the standard *t*-test [28]. All statistical tests were carried out in *R* [29], and made use of the *smatr* package [23], including its common slope test (“*slope.com*”), which compares the fit of a one- and two-slope model to the subdivided data. All code is available on request.

## Results

**Figure 1:**
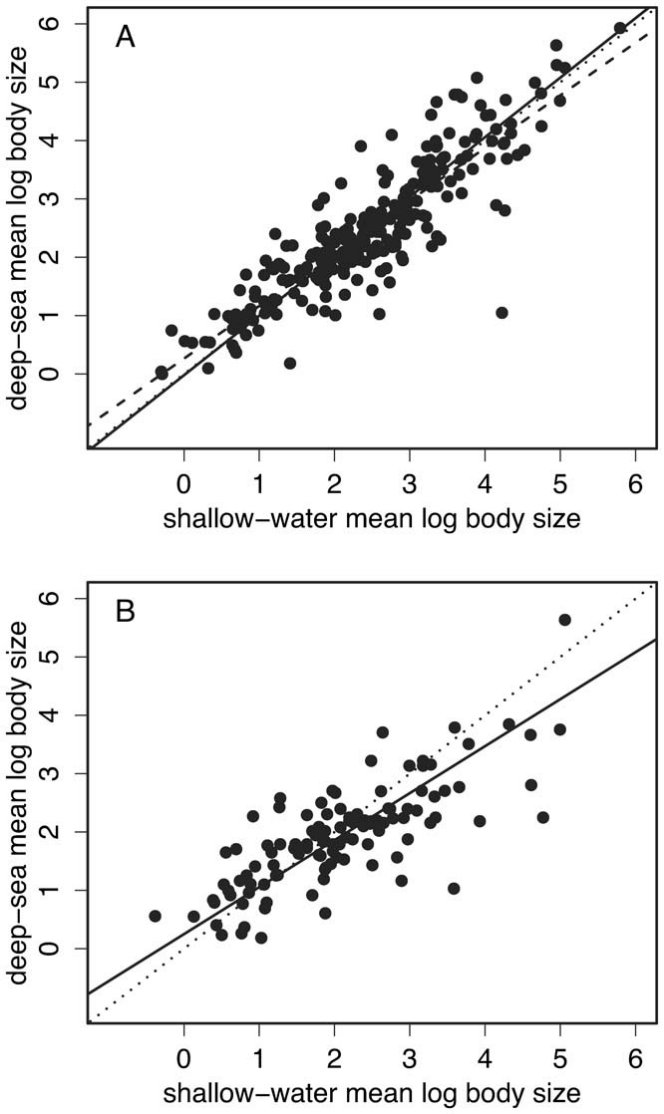
Body sizes of deep-sea gastropods and their shallow-water congeners. Part A shows how different tests of the ‘island rule’ can give qualitatively different results. “Deep-sea” species were defined as those with a depth range midpoint >200m, and all other species defined as “shallow-water”. The ordinary-least-squares regression (dashed line) differs significantly from the 1∶1 line of the null (dotted line), but the standardized-major-axis regression (solid line) shows no significant departure. Part B shows a less ambiguous case: “deep-sea” species are those never observed above 400m, and “shallow-water” species those never observed below 200m; body sizes are within-genus means, taking equal numbers of deep- and shallow-water species in each genus.

To demonstrate the liberal nature of the standard tests of the island rule, consider results when deep-sea habitation is defined via the midpoint of the recorded depth range, i.e., “deep-sea species” have a range midpoint below 200m, and all other species are deemed “shallow-water”. With this definition, 254 genera contained both deep and shallow species, and their generic mean body sizes are plotted in Figure 1A. Applying the standard test [3], [12], the ordinary-least-squares regression slope (dashed line) is found to be highly significantly less than one (*n* = 254; *b* = 0.902; *t*-test p = 0.0015), which offers strong apparent support for the island rule. However, assigning species groups to the “deep” or “shallow” categories at random, showed that even stronger support was obtained with ∼43% of 100,000 randomized data sets, suggesting that there is nothing exceptional in the trend observed in the true data. Accordingly, the standardized-major-axis slope (solid line) was very close to one, and the permutation test showed no significant deviation from the pattern expected if deep-sea colonization had no effect on body size evolution (*n* = 254; *b* = 1.020; permutation *p* = 0.476).

The results above cast doubt on conclusions reached with the standard tests, but they do not refute them. To ask whether an effect really is present, it is better to adopt a more stringent definition of deep-sea habitation [12], Accordingly, let us define as “shallow-water” those species never observed below 200m, and as “deep-sea” those species found only below either 200m, 400m, 600m or 800m. Adopting a range of depth cutoffs is appropriate because the ecological factors responsible for any effect are likely to correlate with depth even below 200m [15], [16], [26]. The data now show strong evidence of the island-rule trend, and the strength and significance of this trend increase steadily with cutoff depth (Table 1 part A(i)). It is possible that these results are an artefact of the use of generic mean body sizes [30]. For example, most genera contain many more shallow-water than deep-sea species (possibly for sampling reasons [31]), and this could bias the regression slope - which is the ratio of the samples' standard deviations [28]. To exclude this possibility, tests were repeated with equal numbers of deep and shallow species per genus (Table 1 part A(ii); Figure 1B), or, as is usual in the literature, with a single species of each type per genus (Table 1 part A(iii)). In both cases, evidence for the graded trend remained, and in some cases became stronger, probably reflecting the preferential inclusion of species with extreme depth ranges (see Methods).

**Table 1:** Deep-sea gastropod body size evolution.

A			(i)		(ii)		(iii)	
	Deep sea	*n*	*b*	*p*	*b*	*P*	*b*	*p*
	>200m	153	0.948	0.203	0.921	0.090	0.908	0.061
	>400m	111	0.862	0.019*	0.805	0.001**	0.832	0.006*
	>600m	74	0.779	0.004**	0.742	0.002**	0.778	0.010*
	>800m	52	0.700	0.001**	0.641	0.000**	0.711	0.008*
B								
	>200m	54	0.837	0.003**	0.797	0.004**	0.819	0.014*
	>400m	42	0.781	0.013*	0.762	0.008*	0.777	0.020*
	>600m	25	0.763	0.020*	0.735	0.020*	0.738	0.068
	>800m	20	0.714	0.010*	0.672	0.011*	0.701	0.041*

Regressions of deep-sea onto shallow-water body sizes. Deep-sea species were defined as those never observed above the depth in the far left-hand column, and shallow-water species as those never observed below 200m. Part A: (i) the mean log body sizes of all species in each genus meeting the depth criteria; (ii) the mean log size of all deep-sea congeners, and an equal number of shallow species, chosen to maximise the difference in midpoint depth range (or vice versa for genera with more deep than shallow species); (iii) the single deep and shallow species with maximal difference in midpoint depth range. *n*: sample size (i.e., the number of genera); *b*: standardized-major-axis regression slope; *p*: *p*-value from 100,000 random permutations of the data.

* *p*<0.05.

** *p*<0.005.

For Part B, genera with fewer than two deep- and two shallow-water species were excluded (so B(iii) uses exactly four species from each genus).

**Figure 2:**
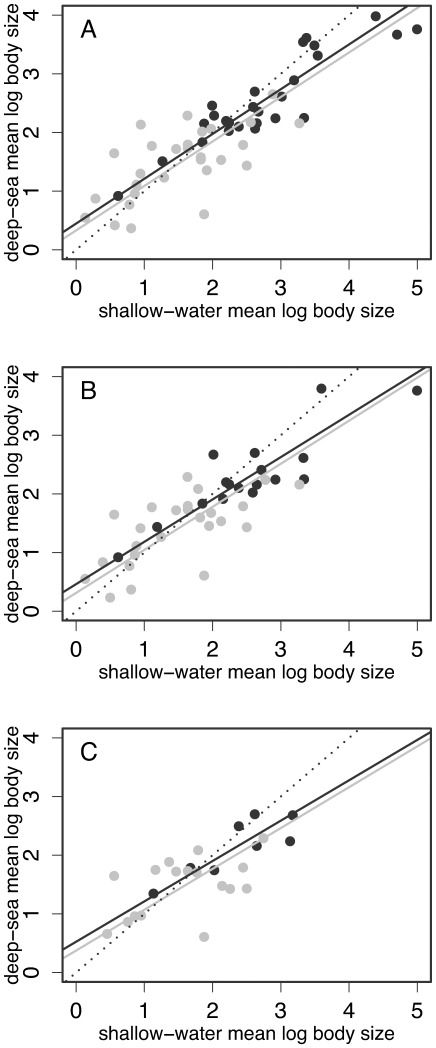
Comparison of effect size across depths and taxonomic groups. The body sizes of deep-sea gastropods are plotted against those of their shallow-water congeners. “Shallow-water” species were never observed below 200m, and “deep-sea” species never observed above depths of A: 200m, B: 400m and C: 600m. Separate standardized-major-axis regression lines are shown for the Neogastropoda (black points) and all other groups (grey points). The dotted line is the 1∶1 expected under the null. Genera with fewer than two deep and two shallow species were excluded.

Averaging across species has untested statistical properties [30], but it does have the advantage of reducing noise and the influence of anomalous data. For example, Figure 1B plots results for balanced samples with “deep-sea” defined as >400m. These data are clearly noisy, and the slope is strongly influenced by a single outlier (the largest value on both axes). This point represents the genus *Fasciolaria*, which contains just a single deep-sea species, the recently discovered *Fasciolaria tephrina* [32]. To restrict the influence of such isolated observations, McClain et al. [12] excluded from their analyses all genera with fewer than two shallow and two deep species. Despite reducing sample size by ∼2/3, this procedure strengthens the observed effect, with a highly significant departure from the null now apparent at the shallowest cutoff depth (Table 1 part B; Figure 2).

These results demonstrate the island rule pattern, but they do not tell us whether it represents a consistent trend across the marine Gastropoda; indeed, the same pattern could arise from clade-specific responses to deep-sea colonization, which are associated with large and small size merely by chance [19]. Phylogenetic tests of this hypothesis were recently introduced [19], but these are difficult to apply to Gastropoda, where much of the phylogeny remains unknown [25], and in any case, can be highly conservative [20]. An *ad hoc* alternative is to test for the consistency of the effect across taxonomic groups. The well-supported clade Neogastropoda (unranked by [25]) is represented by around half of the genera in the present data set, and also contains most of the larger-bodied genera. Figure 2 demonstrates that the regression slopes for this clade alone are very close to those for the remainder of the data, and do not differ significantly at any depth [common slope tests: A *p* = 0.969; B *p* = 0.935; C *p* = 0.971]. Furthermore, evidence for the island rule is similarly strong in both halves of the data [permutation tests: A Neogastropoda: *n* = 28 *p* = 0.049, Others: *n* = 26 *p* = 0.022; B Neogastropoda *n* = 17 *p* = 0.048, Others *n* = 25 *p* = 0.069]. These tests lack power, but are at least consistent with an effect that applies homogenously across the group.

Results above suggest that the strength of the effect observed increases with depth (Table 1; Figure 2), and this is analogous to suggestions that the island rule applies most strongly on the smallest islands, and not at all on the largest islands, whose ecologies can approximate those of continents [4], [6], [18], [27]. In the present context, it is important also to distinguish between different types of deep-sea habitat. In particular, the bathyal zone (including the continental slope) and the deeper abyssal zone differ fundamentally not only from the photic ocean (<200m), but also from each other, both in faunal composition (including body size ranges), and overall ecosystem function [15], [16], [26]. The Malacolog data set includes many species whose ranges are wholly or partly abyssal (defined as deeper than 3000m [26]), and so it is important to ask whether the patterns observed might be solely due to an effect of abyssal habitation. Accordingly, analyses were repeated excluding all species whose observed depth ranges were either wholly or partly greater than 3000m. Table 2 contains two examples where the effect was observed with the complete data set (Table 1), and shows that the exclusion of abyssal species has little effect. Furthermore, the same applies when species from the shallower bathypelagic zone (depths 1000–3000m) were also excluded (Table 2). The island rule pattern of convergence in body size is therefore observed even for species living wholly in the mesopelagic or “twilight” zone of 200–1000m.

**Table 2:** Gastropod body size evolution above the abyssal or bathypelagic zones.

		A			B		
Excluded species	*S*	*n*	*b*	*p*	*n*	*b*	*p*
min. depth>3000m	43	108	0.863	0.026*	54	0.842	0.005**
max. depth>3000m	92	108	0.868	0.029*	54	0.845	0.005**
min. depth>1000m	140	104	0.886	0.066	49	0.848	0.008*
max. depth>1000m	281	99	0.895	0.098	47	0.859	0.014*

Regressions of deep-sea onto shallow-water body sizes, when abyssal or bathypelagic species were excluded. Deep-sea species were defined as those never observed above 400m (part A) or 200m (part B), and shallow-water species as those never observed below 200m in both cases. Part B excludes genera with fewer than two deep and two shallow species. *S*: the number of species excluded from the analysis; *n*: sample size; *b*: regression slope; *p*: permutation *p*-value.

* *p*<0.05.

** *p*<0.005.

## Discussion

This study has confirmed the important findings of McClain et al. [12] that the marine gastropods of the Western Atlantic show a pattern of body size evolution that is analogous to the island rule, with colonists of the deep-sea benthos tending to converge in size in a graded trend (see also [16]). No evidence was found of phylogenetic heterogeneity in the strength of the observed effect, as results for the Neogastropoda alone were indistinguishable from those for the remaining taxa. In contrast, the strength of the effect did increase systematically with range depth, with deeper-sea species showing a stronger tendency to converge in size. Nevertheless, the effect is still apparent in species inhabiting the mesopelagic zone (200–1000m), and so cannot be attributed to unique features of abyssal ecology.

Since the pattern was first identified [1]–[3] the island rule has been explained in a large number of ways [1]–[11]. A powerful method of distinguishing between the competing explanations is to test for the presence of analogous patterns in systems that share some, but not all of the ecological characteristics of island habitats [4], [12], [34]. For example, one putative contributor to the vertebrate pattern is “immigrant selection”, that is, between-lineage differences in the probability of reaching isolated islands, as opposed to differences in survival after colonisation [4], [35], [36]. The colonization of the deep-sea benthos differs clearly and qualitatively from the colonization of islands, and so if it is assumed that the similar patterns of body size evolution reflect a similarity of underlying cause [12], this argues against immigrant selection as a key determinant of the graded trend that is observed in both cases.

Similarly, predator release is a particularly plausible explanation of the vertebrate island rule [1], [4], [6], [11]; this is partly because it can naturally account for both dwarfism and gigantism (by assuming that large and small body sizes evolve as alternative strategies for predator avoidance), and partly because predator release is so clearly implicated in other unusual characteristics of island endemics (such as tameness) [37], [38]. But there is little evidence that reduced predation characterises the deep-sea [12], [14], and indeed there is direct evidence of substantial predation acting on deep-sea gastropods [12], [39]–[41]. The gastropod results therefore argue against the predator release hypothesis as a general explanation of the island rule [12].

So what ecological factor is common to both systems? McClain et al. [12] argue that reduced resource availability characterises both islands and the deep-sea benthos, and that theories invoking resource limitation [1], [3], [4], [6], [9], [11], [12], [37], [42] are therefore the most plausible explanation of the common pattern. While compelling, this argument is not conclusive. First, as recognised by McClain et al. [12] the two environments are characterised by resource limitation of quite different kinds: reduced resources per unit area in the deep-sea (albeit with some probable exceptions such as hydrothermal vents) [12], [15], [42], and a reduced amount of total resources on small islands, but not necessarily reduced productivity per unit area (again, with exceptions such as very young volcanic islands). Second, theories that invoke limitation in the total amount of resources (and that therefore apply to islands) are often clade selectionist, i.e., they assume that we will tend to observe individuals of the size that minimises the extinction risk of their population [9], [37], [43]. Explanations of this kind need to be employed with great care [44], and to explain the island rule, it must be demonstrated that individual selection tends towards the same outcome, or that differential extinction has indeed played an important role. Furthermore, it is not clear that total resource availability is limiting in some well-studied cases [7], [45].

We are therefore still far from understanding the causes of the patterns observed – and particularly the roles of inter- and intra-specific competition [3], [4], [11], [12]. A detailed clarification of where the pattern does and does not hold will be an important step toward achieving this goal [4], [12], [19], [20].

## Supporting Information

Table S1 Supplementary Data Table
(0.29 MB TXT)
